# Unwanted effects: Is there a negative side of meditation? A multicentre survey

**DOI:** 10.1371/journal.pone.0183137

**Published:** 2017-09-05

**Authors:** Ausiàs Cebolla, Marcelo Demarzo, Patricia Martins, Joaquim Soler, Javier Garcia-Campayo

**Affiliations:** 1 Universitat de València, València, Spain; 2 CIBER de la Fisiopatología de la Obesidad y la Nutrición (CIBEROBN), Instituto de Salud Carlos III, Madrid, Spain; 3 Mente Aberta – Brazilian Center for Mindfulness and Health Promotion, Universidade Federal de Sao Paulo, Sao Paulo, Brazil; 4 Hospital Israelita Albert Einstein, São Paulo, Brazil; 5 Hospital de la Santa Creu i Sant Pau & Institut d'Investigació Biomèdica Sant Pau (IIB-SantPau), Barcelona, Spain; 6 Psychiatry and Legal Medicine Department, Universitat Autònoma de Barcelona, Barcelona, Spain; 7 Centro de Investigación Biomédica en Red de Salud Mental (CIBERSAM), Instituto de Salud Carlos III, Madrid, Spain; 8 Hospital Universitario Miguel Servet, Zaragoza, Spain; 9 Instituto Aragonés de Ciencias de la Salud, Zaragoza, Spain; 10 Red de Investigación en Actividades de Prevención y Promoción de la Salud (REDIAPP-G06-170 and RD06/0018/0017), Zaragoza, Spain; Cardiff University, UNITED KINGDOM

## Abstract

**Objectives:**

Despite the long-term use and evidence-based efficacy of meditation and mindfulness-based interventions, there is still a lack of data about the possible unwanted effects (UEs) of these practices. The aim of this study was to evaluate the occurrence of UEs among meditation practitioners, considering moderating factors such as the type, frequency, and lifetime duration of the meditation practices.

**Methods:**

An online survey was developed and disseminated through several websites, such as Spanish-, English- and Portuguese-language scientific research portals related to mindfulness and meditation. After excluding people who did not answer the survey correctly or completely and those who had less than two months of meditation experience, a total of 342 people participated in the study. However, only 87 reported information about UEs.

**Results:**

The majority of the practitioners were women from Spain who were married and had a University education level. Practices were more frequently informal, performed on a daily basis, and followed by focused attention (FA). Among the participants, 25.4% reported UEs, showing that severity varies considerably. The information requested indicated that most of the UEs were transitory and did not lead to discontinuing meditation practice or the need for medical assistance. They were more frequently reported in relation to individual practice, during focused attention meditation, and when practising for more than 20 minutes and alone. The practice of body awareness was associated with UEs to a lesser extent, whereas focused attention was associated more with UEs.

**Conclusions:**

This is the first large-scale, multi-cultural study on the UEs of meditation. Despite its limitations, this study suggests that UEs are prevalent and transitory and should be further studied. We recommend the use of standardized questionnaires to assess the UEs of meditation practices.

## Introduction

Meditation has been used for more than a thousand years as a spiritual or healing practice. It encompasses a set of practices that can be subdivided into three umbrella groups: attentional (as in mindfulness meditation), constructive (as in compassion or loving kindness meditation), and deconstructive (as in object-oriented insight) [1]. In the past 20 years, in Western societies, there has been an increase in the use of meditation practices related to mindfulness-based interventions (MBI), one of the most frequent meditative approaches used in the scientific context. It has been well documented that meditation and MBIs are associated with several positive outcomes, such as a decrease in psychiatric symptomatology (e.g., anxiety, depression) or an increase in positive states (e.g., well-being, positive emotions) [2,3]. Furthermore, changes in brain areas involved in positive health have been reported [4]. However, despite the long-term use of meditation and its evidence-based efficacy in diverse clinical, educational, sport, and industry applications, there is still a lack of data about the possible unwanted effects (UEs) of these practices.

The research on side effects and UEs of health practices has been developed to a large extent in the context of pharmacological interventions that can lead to the onset of mild, moderate, or severe adverse effects [5]. These effects are usually transitory and sufficiently severe that they may eventually lead to discontinuing treatment, or even to hospitalization or death.

In the field of psychological treatments, which can include meditation programmes and practices, UEs of psychological interventions are rarely assessed, although some ad hoc questionnaires have been developed [6–11]. In the area of meditation practices and MBIs, scientific studies on UEs are uncommon [12, 13] and usually based on case reports or speculative hypotheses. Additionally, most of them were conducted years ago [14– 17]. Other studies have indicated that long meditation periods can be contraindicated for people with psychiatric problems [18], as they promote the precipitation of mental illness and psychosis [19]. Among the aforementioned reactions, the practice of meditation may have associated psychological (e.g., emotional stress, confusion, disorientation, and dependence on practice), psychopathological (e.g., anxiety, deliriums, and hallucinations), and physiological (e.g., pain, sensorial dysfunction, exacerbation of neuromuscular/joint diseases, reduction in appetite and insomnia) symptoms [20]. The most severe cases reported include the precipitation of psychosis [14], posttraumatic stress disorder (PTSD) [20, 21], and epileptic attacks [22].

Regarding MBIs, Randomized Control Trial studies do not usually report possible UEs, and therefore we have no information about their prevalence [13]. MBIs involve low intensity practice compared to traditional meditation practices, and meditation sessions in MBIs are generally short and spaced far apart. Because there is a higher probability of UEs with increased practice time [16], it is assumed that these reactions tend to be less frequent and/or intense in MBIs than in so-called traditional intensive meditation practices, such as those found in Buddhism. Another interesting topic is the relationship between different meditative practices and UEs; as previously reported, meditative practices can be subdivided into three umbrella groups: attentional, constructive, and deconstructive [1]. However, there are no previous results about these practices and the frequency of UEs.

It should be noted, however, that even in Buddhist practices, UEs tend to be relative, with psychological and physiological changes often considered to be natural and inherent to the spiritual process [23, 24]. On the one hand, the lack of knowledge about experiences related to traditional meditation practices, detaching them from a religious or cultural context, can result in their misinterpretation as unpredictable events and their misdiagnosis as physiological or psychological disorders resulting from the practice or UEs [25].

This gap in the knowledge could be explained by the lack of standardization in reporting harm [20, 26]. In addition to these possible flaws, factors that limit MBI trials involving UEs include the difficulty associated with obtaining a statistically significant number of participants and the type of design of these studies [6, 27, 28]. Research on UEs of meditation practices and MBIs is relevant because there is still a lack of data on possible harm and the types of people who may not benefit from these types of practices. As Josipovic and Baars [29] reported, this research is an ongoing challenge in contemplative practices.

The aim of this study was to evaluate the occurrence of UEs in meditation practitioners, considering moderating factors such as the type, frequency, and lifetime duration of the meditation practices. Our initial hypotheses were as follows: 1) The study will find a limited number of UEs that will be mild and transitory; 2) UEs will be more frequent when there is a higher volume of practice; and 3) some types of meditation practices will be more prone to generating UEs.

## Methods

### Participants and data collection

An online survey was developed on a commercial system (www.surveymonkey.com) and disseminated through several websites, such as Spanish-, English- and Portuguese-language scientific research webpages related to mindfulness and meditation, local and nationwide mindfulness associations, Zen monasteries, and different meditation groups or *sangha* websites. Participants were invited to participate in research to “study the occurrence of certain unexpected (not normally expected) and/or unwanted effects during the practice of meditation or mindfulness”. The link was accessible from April 2013 to July 2015. Overall, 914 (686 Spanish-, 59 Portuguese-, and 123 English-speakers) participants accessed the link, and 908 voluntarily agreed to participate. Participants who did not answer the survey correctly or completely (n = 509) and those who had fewer than two months of meditation experience (n = 57) were excluded. According to most MBIs, two months is the minimum time needed to learn to meditate. Ultimately, 342 participants were included in the study. The study was approved by the Aragon Ethics Committee, and all participants provided written informed consent prior to their inclusion in this study.

Participants were asked to report: sociodemographic data; previous health problems, such as anxiety and depression (Are you experiencing anxiety symptoms? Are you experiencing depressive symptoms?); type of meditation practice (focused attention (FA), open monitoring (OM), body awareness (BA), compassion (C), imagination (I), and informal practices (IP)); frequency and lifetime practice of meditation; and the learning context (self-taught (e.g., books, Internet), teacher (religious context: Buddhist, Christian, etc.), in the context of therapy, secular teaching or a training course (non-religious context)). Regarding the UEs, participants were first asked about the possibility “of having experienced any type of unwanted (not normally expected) or adverse reactions (with potential harm to your health) resulting from the practice of meditation” (“yes” or “no” options). If the answer was “yes”, using a qualitative approach with an open question, they were asked to describe in detail the reactions, the context where they occurred (retreat, individual, or group), the type of meditation practice, and the length of the session. Moreover, participants (regardless of whether they answered “yes” or “no”, n = 276) were invited to complete a checklist of 18 different potential experiences obtained from the literature [20] to explore UEs that were not reported or missed due to memory bias. Responses for occurrences ranged from 0 (“never”) to 10 (“frequently”).

## Results

The sociodemographic profiles of the study participants are shown in Table 1. The majority of practitioners were women (68.4%), from Spain (42.9%), married (48.5%), and had a university education level (54.4%). Regarding the frequency of meditation practices evaluated in the survey (Table 2), informal practices were more frequently reported on a daily basis (46.8%), followed by focused attention (30%), whereas compassion practices were practiced the least. Moreover, 16.6% (n = 52) of the participants reported depressive symptoms, and 23.4% (n = 75) reported anxiety symptoms.

**Table 1:** Sociodemographic data for the sample.

		*M*	SD
**Age**		39.7	(12)
		**TOTAL**	**%**
**Gender**	Men	108	31.6
	Women	234	68.4
**Nationality**	Spain	145	42.9
	Argentina/Uruguay	28	8.3
	Mexico	34	10.1
	Colombia	15	4.4
	Rest of Europe	11	3.3
	Brazil	22	6.5
	Peru	3	.9
	Chile	6	1.8
	Venezuela	6	1.8
	United Kingdom	20	5.8
	USA & Canada	25	7.3
	Other countries	23	6.7
**Marital status**	Single	122	35.7
	Married	166	48.5
	Widowed	7	2
	Divorced	46	13.5
**Education level**	Primary	4	1.2
	Secondary	10	2.9
	Higher secondary	28	8.2
	University	186	54.4
	Master and/or PhD	114	33.3

**Table 2:** Type, frequency (%) and years of practice (mean and standard deviation).

	Daily	3-4x per week	1x per week	1x per month	Never	Years of practice
**FA**	103 (30%)	87 (25.4%)	50 (14.6%)	36 (10.5%)	66 (19.3%)	**4.9 (5.7%)**
**OM**	71 (20.8%)	66 (19.3%)	28 (8.2%)	37 (10.9%)	140 (40.9%)	**5.6 (7.2%)**
**I**	32 (9.4%)	27 (7.9%)	41 (12%)	65 (19%)	177 (51.8%)	**5.2 (5.9%)**
**BA**	46 (13.5%)	83 (24.3%)	46 (13.5%)	40 (11.7%)	127 (37.1%)	**6 (6.6%)**
**C**	41 (12%)	32 (9.4%)	30 (8.8%)	43 (12.6%)	196 (57.3%)	**5.9 (8.6%)**
**IP**	160 (46.8%)	38 (11.1%)	**18 (5.3%)**	**13 (3.8%)**	**113 (33%)**	**5 (7.3%)**

Note: FA = Focused attention; OM = Open monitoring; I = Imagination; BA = Body awareness; C = Compassion; IP = Informal practice

Of the participants, 25.4% reported UEs (Table 3), showing that severity varies considerably. Most of the reported events were transitory (39%), and most of them did not lead to discontinued meditation or the need for medical assistance. UE occurrence was more commonly reported in relation to individual practice (41.3%) (as opposed to retreat or group practices), during focused attention meditation (17.2%), and when practising for more than 20 minutes (20.6%). All descriptions were categorized by three experts into eight pre-established categories using content analysis: Anxiety symptoms (including panic attacks “During a meditation a painful image came to my mind, and my heart started to beat fast, and I started to feel fear and panic”); Pain (stomach, headache, muscular, nausea) (“Extreme back, shoulder, and neck muscle pain”); Depersonalization and derealisation (“when I began to focus on my breath, a shift seemed to occur in my spatial awareness quite quickly. I felt like my awareness was becoming very close to me, and everything around me was becoming very distant.); Hypomania or depressive symptoms (“Feelings of being over-energized and an inability to sleep”); Emotional lability, Visual focalization problems, Loss of consciousness or dizziness (“Feeling of falling into a void”); and non specific information. The rate of agreement was 95%. The most frequently described reactions were anxiety symptoms (13.7%) and depersonalization or derealisation (8.0%) (S1 Table). UEs were not related to anxiety (X^2^ = .144) or depressive (X^2^ = .245) symptoms.

**Table 3:** Percentage of self-reported UEs and characteristics (n = 87).

		n	%
**Unwanted effects (UEs)**	Yes	87	**25.4**
	No	255	**74.6**
**Situation**	Individual practice	36	**41.3**
	Retreat	12	13.7
	Group (non-retreat)	9	10.3
	No information	30	34.4
**Type of practice**	Body scan	7	**8**
	Focused attention	15	17.2
	Open monitoring	8	9.1
	Mantra	7	8
	Awareness of emotions	1	1.1
	Yoga (pranayama)	2	2.2
	No information	47	54
**Duration of the meditation session (min)**	>40	9	**10.3**
	21–40	9	10.3
	16–20	1	1.1
	5–15	4	4.6
	No information	64	73.5
**Symptoms**	Anxiety symptoms (including panic attacks)	12	**13.8**
	Pain (stomach, headache, muscular, nausea)	5	5.7
	Depersonalization and derealisation	8	**9.2**
	Hypomania or depressive symptoms	2	2.3
	Emotional lability	2	**2.3**
	Visual focalization problems	2	2.3
	Loss of consciousness or dizziness	6	**6.9**
	Other symptoms	4	**4.5**
	No information	46	**52.8**
**Continuous or transitory**	Continuous	9	**10.3**
	Transitory	34	39
	No information	44	50.5
**Discontinued meditation**	Yes	11	**1.1**
	No	33	37.9
	No information	43	49.4
**Assistance from a medical centre or specific therapist**	Yes	5	**5.7**
	No	39	44.8
	No information	43	49.4

A Kruskal-Wallis non-parametric test was used to analyse the differences between UEs and frequency of practice and years of practice. Regarding frequency, the results showed that the only significant difference was related to body awareness practice (*X*^*2*^ = 5.335; p <.05), with fewer UEs being found in the group that practised more frequently. With regard to years of practice, no significant differences were found. Furthermore, a Chi square analysis was conducted to test the differences between contexts of learning (X^2^ = .434) and sociocultural background (X^2^ = .840), with no significant results found.

The UE structured checklist was completed by 276 participants (including those who reported no previous UEs) (Table 4). The most common experiences (based on the number of people with the maximum score) were “feelings of being alienated from society” (4.6%), “difficulty in feeling comfortable in the world” (4.2%), and “feeling that something is lacking” (4.2%).

**Table 4:** Frequency of reporting unwanted effects of meditation from a checklist.

	X (SD)	% max	% min
Greater emotional pain	3.3 (3)	2.7	52
Greater self-criticism	3.7 (3.2)	3.1	45.6
More fear/anxiety/depression	2.7 (2.8)	3.1	59.8
Lack of life orientation	2.5 (2.7)	3.1	68.1
Less motivation in life	2 (2.3)	1.5	76.7
Feeling of needing something more, of lacking something, etc.	3.1 (3.1)	4.2	58.8
Greater mental confusion	2 (2.3)	.9	74
Lack of interest in your surroundings	2.4 (2.6)	1.2	66.7
Boredom	2 (2.2)	1.2	73.4
Need for continuous meditation	2.7 (2.7)	1.5	54.5
Feeling that the time not spent meditating is wasted	2.1 (2.1)	.4	67.2
Restlessness/anxiety when not practising formal meditation	2.6 (2.4)	.8	56.5
Increased criticism of others	2.4 (2.4)	1.5	60.7
Greater awareness of their negative traits	3 (2.8)	2.3	52.3
Feeling of being superior to them/better than them	2.2 (2.2)	.4	62.9
Feelings of boredom caused by people	2.4 (2.5)	1.9	61.7
Feelings of lack of interest in others	2.1 (2.2)	.8	69
Lack of interest in people’s conversations	2.5 (2.6)	2.3	66.3
Feeling that only people who meditate are valuable	1.8 (1.7)	.4	71.6
Feeling of being alienated from society	2.8 (2.9)	4.6	59.9
Hypersensitivity/rejection of urban life	3.3 (2.9)	3.8	44.6
Difficulty in feeling comfortable in the world	2.8 (2.8)	4.2	58.6

Note: % max = Percentage of participants with the maximum score (10).

Finally, a univariate ANOVA test was performed to find out whether there were differences in the checklist related to the learning contexts. The results showed significant differences in “greater emotional pain” [F(3,227) = 2.908; p <.05; *η*^2^ = .037] and the “need for continuous meditation” [F(3,225) = 2.793; p <.05; *η*^2^ = .036]. The Bonferroni post hoc analysis showed significant differences between the self-taught [M = 2.6; SD = 2.7] and teacher context [M = 4.3; SD = 3.2] (p <.05), as well as the self-taught [M = 3.5; SD = 3.2; p <.05] and secular teaching or training course [M = 2.9; SD = 2.7] (p <.05).

## Discussion

To the best of our knowledge, this is the first cross-sectional correlational study addressing UEs of meditation across different countries with a large number of respondents. Previous studies consisted of narrative case descriptions of side effects or surveys with small samples [16, 22, 30].

We found a high percentage of reported UEs (25.4% of respondents), although it must be emphasized that a great deal of essential information was lost because many participants (approximately 50%) did not provide complete details about the episodes. Curiously, this issue is not addressed in the main mindfulness protocols (MBSR, MBCT) or in most studies on MBIs. It seems that the expansion of mindfulness in the West has been associated with a gentle, positive vision of the technique, without the necessary balance related to the negative consequences of any practice. Most reported UEs are mild and do not lead to discontinued meditation or the need for medical assistance, as described in previous papers [31, 32], which may have contributed to the lack of research about UEs, but trainers and practitioners should be aware of these effects. Furthermore, there is a lack of knowledge about the presence of UEs because meditation practice can be “extremely psychologically challenging” [33]. In fact, EUs can be related to changes in a detached perspective of the self [34], different stages of insight [33], or the use of meditation in participants with previous pathological disorders that have not been correctly diagnosed [13].

The study also confirmed our second hypothesis that a higher frequency of practice may generate more UEs, especially in terms of the duration of the session. In addition, UEs seem to be more frequent during individual practice than during group practice. There are no studies on this subject, but meditators’ experiences suggest that long meditation sessions are more prone to producing UEs, whereas group practice seems to be easier and more reinforcing for many meditators. Prospective studies are necessary to weigh the percentage of total meditation time devoted to long sessions and group practice because it is possible that these findings are simply explained by the fact that most meditation time is made up of individual long sessions.

Our findings also confirmed our initial hypothesis that some types of meditation practices may interact to influence the prevalence of UEs. The most relevant data show that focused attention practices are associated with more UEs, whereas body awareness practices are associated with fewer UEs. Body consciousness has been described as one of the aetiological mechanisms of mindfulness [34, 35, 36], and it has been found to be related to psychological and medical health [37]. Body awareness, along with detachment and acceptance of emotions, has been linked to the associations of meditation and mindfulness with depression and anxiety [38]. Focused attention is described in the tradition as the first step in meditation, but Buddhism refers to it as a source of extreme views [39].

Finally, when a checklist of potential UEs was presented to respondents, although the scores were not high, there was a percentage of people with the maximum score. Regarding the relationship with the practice, FA was found to be positively associated with “greater self-criticism” and “feeling that the time not spent meditating is wasted”, although other types of mediation protected against some potential UEs, especially imagination practices. FA procedures emphasize bringing one’s attention to a unique and particular object of meditation. Thus, they are more structured and rely on instructions to a greater extent than other types of meditation. Heavily structured techniques may easily lead to a process that conflicts with what the meditator is experiencing [40].

Based on our findings and those presented in previous reports, it is possible to say that UEs can consist of a range of physiological, psychological, psychopathological, and “spiritual” events [16, 18, 19, 22, 25]. However, there is no consensus about whether the majority of these symptoms are directly related to the practice (side or adverse effects, treatment reactions, malpractice effects, and contraindications), or can be considered inherent to it, or simply facilitate the emergence of undiscovered mental or physical problems (adverse events) [10]. In the future, longitudinal trials should address these questions in a more systematic and in-depth way.

The study has several limitations. One of them is the selection of the sample, which was not representative of the meditating population and was recruited over the Internet. There are biases associated with this form of recruitment. In addition, the questionnaires involved self-report and were not previously validated. Another limitation was that the protocol was rather long; in fact, 50% of the individuals who accessed the link did not complete the survey. Furthermore, many participants who reported having UEs did not provide information about the characteristics of the episode. Therefore, there might have been a predominance of people who wanted to share their experiences, both positive and negative. Consequently, extraversion or other personality traits may have had some influence on the responses. The sample was also culturally unbalanced, with a higher prevalence of Spanish and Latin-American participants. In addition, social acquiescence can also produce a bias. Some UEs of meditation are not only physical or psychological, but can also be related to values, spiritual beliefs, and deeply rooted thoughts that the participant may be reluctant to share. Furthermore, in several traditions, UEs are part of spiritual development; therefore, some meditators might view the reactions as normal, or even positive, and not report them as UEs [33]. Finally, memory bias is another limitation. Most of these effects probably occurred months or years before, and many may be forgotten or distorted by memory. The only way to overcome this bias is by using prospective studies, but the sample size and long follow-up period make them expensive and difficult to implement.

## Conclusion

This is the first large-scale, multi-cultural study on the adverse effects of meditation. Despite the study’s limitations, results suggest that UEs are prevalent but transitory, are more frequent in long individual sessions, and have clear associations with various types of meditation. In the future, research on UEs should be incorporated into efficacy studies of MBIs and interventions based on contemplative practices.

We suggest that standardized questionnaires should be used to assess the UEs of meditation in future trials. These questionnaires could be a compulsory safety outcome in MBI evaluation studies, in the same way that questionnaires such as the UKU [41] are used in pharmacological studies to assess safety.

### Public health significance statement

The use of meditation practices related to mindfulness-based interventions has increased, but there is a lack of knowledge about their potential unwanted effects. In this study, 25% of the participants reported unwanted effects of the practice, with anxiety episodes being the most frequent.

## Supporting information

S1 Table Content analysis of the unwanted effects. (DOCX)
