# Supplementary material for: Unwanted effects: Is there a negative side of meditation? A multicentre survey
Source: PLoS One. 2017 Sep 5;12(9):e0183137. doi: 10.1371/journal.pone.0183137 (PMC5584749; doi:10.1371/journal.pone.0183137)
Supplement: S1 Table — (DOCX) [file pone.0183137.s001.docx]

**S1 Table 1. Content analysis of the unwanted effects**

|  | H | Feelings of being over-energize and an inability to sleep. Symptoms of hypomania followed by a manic episode, and a subsequent depressive episode |
| --- | --- | --- |
|  | L | Mareo, sudor por todo el cuerpo, ganas de vomitar |
|  | D | Sensación de irrealidad, de pérdida de la lucidez y de la identidad, pero sin perder la conciencia, intenso miedo, sensación de que el cuerpo se disuelve en el vacío expandiéndose en todas direcciones, con cierta sensaciones de vértigo y nausea. |
|  | P | Nauseas |
|  | A | Opresión torácica y sensación de falta de aire |
|  | L | Desvanecimiento |
|  | V | Visión borrosa |
|  | EL | Más sensibilidad en las sensaciones afectivas en el entorno familiar |
|  | L | Sensación de caer en el espacio. Mareo. |
|  | D | En una ocasiona me encontré como que había abandonado mi cuerpo y estaba flotando viéndome a mí mismo acostado . |
|  | P | Se me reventaron las venas de los tobillos en ambos pies.. |
|  | A | En un retiro, en las meditaciones largas, me dio taquicardia |
|  | A | Con mucha intensidad de mantras, por un período se ha incrementado un poco la ansiedad |
|  | A | Ataque de pánico |
|  | D | When I begin to focus on my breath, it is as if there is a shift in my spatial awareness occurs quite quickly. I feel as though my awareness is becoming very close to myself and everything around me is becoming very distant. |
|  | A | Durante la sentada meditativa vino a mi mente un recuerdo doloroso. Mi cuerpo se encogió, mi pecho empezó a agitarse como consecuencia de mi taquicardia y la relación entre mi pensamiento y mi cuerpo hacía que esa idea se mantuviese viva. En alguna ocasión la angustia hacía que no pudiera respirar y estar casi al borde del ataque de pánico, normalmente en la noche. |
|  | A | Estaba sentado meditando en un retiro de dos semanas. En un momento, me vino un recuerdo doloroso del pasado. Durante la sentada me venía a la mente la posibilidad de perder el control y volverme loco. Sentía taquicardias y encogimiento de todo mi cuerpo y mucha ansiedad. |
|  | D | I was in bed. I could not feel the sheets underneath me. I took a few breaths a d stayed with the feeling. I chose to move my right foot slightly, then I could feel sensations like normal. |
|  | V | Visão ficou totalmente desfocada. Parecia que eu tinha 20 graus de miopia. Após mais uns 25 min essa visão desfocada passou. |
|  | H | Recurrence of depression symptoms which had been at bay for several years |
|  | A | Sensación de nerviosismo, ansiedad, estrés, falta concentración transcurridas unas horas desde la meditación |
|  | A | Anxiety crisis |
|  | D | Sentí que me elevaba |
|  | P | Alteración en la digestión, pequeñas punzadas en el estómago y una pequeña diarrea. |
|  | L | Adormecimiento de la lengua Sueño extremo, casi desmayo |
|  | L | Cuando ya estaba en el punto, hubo como una especie de interrupción eléctrica interna y al despertar estaba casi inconsciente |
|  | A | Ataques de ansiedad, con palpitaciones, taquicardia, sudores. Ataques de pánico con sensación de muerte inminente y sensación de pérdida de control de la realidad. Pesadillas con sensación de muerte. |
|  | EL | Más sensibilidad en las sensaciones afectivas en el entorno familiar ante, separaciones físicas o contactos telefónicos con ellos (esposa, hijos etc.) |
|  | A | Conseguí soltar partes del cuerpo que estaban tensas y desencadenó unos días de ansiedad hasta que se volvieron a normalizar |
|  | D | Sentí como si me estuviese viendo desde fuera |
|  | P | Extreme back, shoulder, and neck muscle pain. Severe headache. |
|  | L | Pérdida de la voluntad sobre algunas partes de mi cuerpo. |
|  | A | Empecé a sentir que perdía el control y que me podía volver loco. Físicamente sentía una fuerte sensación de angustia en el pecho con un poco de taquicardia y sensación de mucho miedo. |
|  | D | La percepción de mis sensaciones corporales cambiaron. Me Noté más sutil flotando sobre mi cuerpo físico. Escuché ruidos/zumbidos me vi entrando en un túnel oscuro viajando a mucha velocidad |
|  | A | Angustia y ansiedad |
|  | D | Mareo y sensaciones de vértigo o desratización. |
|  | O | Tuve una visión sobre el futuro |
|  | P | Dolores lumbares |
|  | O | Percibía los objetos de forma diferente, tenían un color más brillante |
|  | O | Divagar demasiado sobre si hacía bien las prácticas, e intentar racionalizar todo lo que pasaba. |
|  | O | Convulsiones en el cuerpo |

Note: A= Anxiety symptoms (including panic attack); P= Pain (stomach, headache, muscular, nauseas...); D= Depersonalization and derealization; H= Hypomania or depression symptoms; EL= Emotional lability; V= Visual focalization problems; L= Loss of consciousness or dizziness; O= Others.
